# Applications of Shape Memory Alloys for Neurology and Neuromuscular Rehabilitation

**DOI:** 10.3390/jfb6020328

**Published:** 2015-05-27

**Authors:** Simone Pittaccio, Lorenzo Garavaglia, Carlo Ceriotti, Francesca Passaretti

**Affiliations:** 1 National Research Council of Italy, Institute for Energetics and Interphases (CNR-IENI), C.so Promessi Sposi, 29-23900 Lecco, Italy; E-Mails: l.garavaglia@ieni.cnr.it (L.G.); c.ceriotti@ieni.cnr.it (C.C.); f.passaretti@ieni.cnr.it (F.P.); 2 Politecnico di Milano, P.za Leonardo da Vinci, 20133 Milano, Italy; E-Mail: lorenzoelia.garavaglia@polimi.it

**Keywords:** pseudoelasticity, neurologic disorders, movement disorders, neuromuscular rehabilitation, orthoses, robotic rehabilitation, spasticity

## Abstract

Shape memory alloys (SMAs) are a very promising class of metallic materials that display interesting nonlinear properties, such as pseudoelasticity (PE), shape memory effect (SME) and damping capacity, due to high mechanical hysteresis and internal friction. Our group has applied SMA in the field of neuromuscular rehabilitation, designing some new devices based on the mentioned SMA properties: in particular, a new type of orthosis for spastic limb repositioning, which allows residual voluntary movement of the impaired limb and has no predetermined final target position, but follows and supports muscular elongation in a dynamic and compliant way. Considering patients in the sub-acute phase after a neurological lesion, and possibly bedridden, the paper presents a mobiliser for the ankle joint, which is designed exploiting the SME to provide passive exercise to the paretic lower limb. Two different SMA-based applications in the field of neuroscience are then presented, a guide and a limb mobiliser specially designed to be compatible with diagnostic instrumentations that impose rigid constraints in terms of electromagnetic compatibility and noise distortion. Finally, the paper discusses possible uses of these materials in the treatment of movement disorders, such as dystonia or hyperkinesia, where their dynamic characteristics can be advantageous.

## 1. Introduction

Neuromuscular disorders are a large group of diseases, which affect the central nervous system (CNS). As a consequence, the ability to move and control one’s limbs in voluntary actions is affected, resulting in disability, loss of quality of life and functional independence. 

Among the neuromuscular disorders, this paper will address those that depend on upper motorneuron lesions (UML; or lesions of the cortico-spinal tract) of various etiologies (stroke, traumatic brain injury, arteriovenous malformations, anoxia, *etc.*) and some of the movement disorders (MD; e.g., tremors, dystonia, dyskinesias) caused by alterations in the normal behavior of the basal ganglia [1]. The former share similar motor symptoms, among which the most relevant are a complete or incomplete paralysis of different areas of the body, with rigidity, muscular contractures, hypertone and spasticity; the latter display a phenomenology that is more varied, but is often characterized by the insurgence of phasic or tonic involuntary movements of different extent, duration, frequency, location and direction, as well as abnormalities of posture. The basal ganglia (striatum, pallidum and related structures, including the substantia nigra and subthalamic nucleus), in fact, together with the cerebellum, play an important role in the control of muscle tone, posture and coordination of movement by virtue of their connections, via thalamo-cortical fibers, with the cortico-spinal system and other descending cortical pathways.

Physical rehabilitation is fundamental in promoting the recovery of lost functions in all of these neurological disorders [2]. Although diagnosis and disease etiology are important and can inform about the expected range of problems and natural history of each illness, individual manifestations of disability are as fundamental in defining the needs of patients during rehabilitation. Therapy is often multimodal, including manipulation by physical therapists, orthotics, robotic therapy, active exercise and the use of drugs. All of these actions, together, aim at restoring the normal characteristics of muscles and joints, their voluntary control by the patient, segmental and task-oriented functionality and, ultimately, personal and social independence. The relative weight of each mode of treatment is strongly dependent on the clinical conditions of single patients, so that the overall plan must be customized. Furthermore, there may be issues affecting the choice and efficacy of some of the standard therapeutic protocols, such as individual adverse responses to drugs, intractability of some districts (e.g., due to pain), limited time for man-delivered physical therapy and limitations in the use of robotic devices due to patients’ specific impairments.

It can be of particular interest to focus on the development of new devices that support physical treatment in neurology and neuromuscular rehabilitation, because innovative devices can help expand the set of treatment approaches now available, allowing more patients with peculiar characteristics to be treated and limiting the inapplicability of some types of therapy due to general or individual adverse effects. This paper will discuss wearable devices (orthoses) for the quasi-static and dynamic repositioning of affected body segments. The application of this type of treatment is quite broad and widespread in the case of spastic UML-derived syndromes, because fighting the insurgence of ill-postures and their becoming chronically structured is essential for safeguarding the possibility of functional recovery; orthotics have currently a more limited use in dynamic movement disorders, even though for some of them (e.g., tremor and dystonia), this type of device can be employed, especially when other approaches are proven useless. Some of the problems connected to the practical application of orthoses in treating of MD patients depend on the fact that they often do not possess sufficient characteristics in terms of aesthetics (appearance, size and shape), cosmetics and functionality for people with a social and more-or-less independent daily life [3]. Another topic, which will be considered in this paper, is the use of passive limb mobilization as a means of both acting positively for the maintenance or recovery of tissue properties in UML and providing continual proprioceptive and somatosensory afference to the brain during the paretic phase of the disease (especially in the early period after the acute event). While the importance of robotic devices for intensifying physical therapy and promoting better functional recovery has been recognized in the past [4,5], the analysis of its cortical effects is still underway. In order to be able to investigate this neuroscience correlate of rehabilitation treatments, it is advantageous to have available a set of robotic devices for limb mobilization that are compatible with the tight electromagnetic constraints that modern diagnostic instrumentation (e.g., magnetic resonance imaging, magnetoencephalography, electroencephalography) impose; this subject will be discussed, too.

Versatility and customization can be valid starting points to construct new devices for the afore-mentioned applications. Neurological insults generate a loss of function, which is related to the specific location of the primary damage. On the other hand, the appearance of later sequelae can be due to natural history or to the missing or delayed tackling of specific problems. For instance, in the case of UML syndromes, the immobility and disuse caused by acute paresis and the possible lack of early mobilization can determine the onset of vicious circles leading to muscular contractures and spasticity. Recognizing that the conditions of patients are not just characterized by individual components, but are also in continuous evolution, is a sound basis for imagining new devices that address patient-specific phenomenology, try to bridge between early and later phases of the disease and anticipate and prevent negative developments. Therefore, new patient-aware devices should be characterized by properties that adapt to changing patient-specific features, in the most physiological way. 

The use of conventional materials may pose strong limitations in versatility (in particular for wearable orthoses), due to the fact that material characteristics are fixed and do not follow very well the dynamic changes in patient’s clinical needs or disorder evolutions. This can be true, to some extent, also for other types of devices, as will be explained. Materials with unusual and nonlinear properties can hence be investigated as possible alternatives to standard ones. A class of compounds that display interesting characteristics in terms of deformability, strength, weight and reliability, which are therefore good candidates for biomedical applications, is shape memory alloys (SMAs) [6]. A satisfactory balance between these properties gives the SMA materials the right characteristics to be employed in a number of different fields and, in particular, those related to physical rehabilitation. Among the several properties of SMA, pseudoelasticity and the shape memory effect (SME) are the most useful in neurology and neuromuscular rehabilitation applications: in particular, stable (quasi constant stress levels) and long (large deformability ranges) plateaux and also the possibility to modify those parameters with thermomechanical treatments can be exploited in designing a variety of devices and solutions for rehabilitation; because, in addition, these same materials also display interesting internal friction and mechanical hysteresis characteristics, the peculiarities of SMA could be of help in applications that possess dynamic characteristics.

There are a number of groups who have been developing ideas and devices exploiting SMA for imparting forces on or producing movements of body parts, especially with the aim of assisting or replacing lost functions. The most relevant experiences address biomedical problems, such as the mobilization of paralyzed hands, fingers (e.g., [7,8,9]) and other segments [10], the support of gait (e.g., [11,12,13,14,15]) and limb repositioning [16]. The corpus of published literature highlights the promising aspects of SMA technology [17] and also describes the limitations connected with those materials. Considering the designs based on the shape memory effect, most papers mention the compactness and the possibility to develop flexible technologies as valuable aspects of SMA actuation, while the trade-off between torque or force output and actuation speed appears to be the main issue in rehabilitation applications. The question of actuator control, which is also one of great consequence, is approached by various authors in different ways, depending on the final aim: the literature on rehabilitation applications of SMA describes open-loop strategies directed at simply triggering the start or pacing the repetition of actuation cycles [10,11]; open-loop methods are also proposed to obtain specified movement trajectories [8,9]; alternatively, more sophisticated closed-loop strategies are reported to provide very precise control of actuation timing and output parameters [18,19].

The use of pseudoelasticity is proposed in different papers tackling, in particular, limb positioning and gait rehabilitation [14,15,16]. In those works, the deformability, adaptability and the nonlinear mechanical properties of SMA are considered as a resource for obtaining compliant and biomechanically-sound solutions to the clinical problems connected with spasticity and paresis. The main aspects considered in the optimization of those devices are the tuning of alloy properties and the characterization/modeling of their thermomechanical behavior in order to provide appropriate static or dynamic corrective torques.

An additional review of prior results can be found in [20].

The present work will show not only additional specific examples of designs that employ SMA in rehabilitation applications, but will also try to establish a link between the properties of these materials and the development of innovative therapeutic approaches for neuromuscular disorders focusing on the exploitation of residual patients’ capabilities and interacting dynamically with their evolving conditions.

## 2. Results and Discussion of Selected Rehabilitation Applications

In relation to the characteristics discussed above, some applications of SMA in the field of neuromuscular rehabilitation will be reported and described, with the aim of demonstrating the feasibility and relevant outcomes and showing how the different functionalities of this class of materials can be applied.

### 2.1. Portable Devices for Passive and Aided Exercise 

The physical rehabilitation of patients that suffer from paresis as a consequence of a neurological insult generally includes active exercise, which is often segmental at an initial stage and becomes increasingly functional as motor recovery proceeds. Although it is recognized that active exercise is extremely important for the re-acquisition of motor skills, passive mobilization of the limbs is also a standard part of physical treatment, because it can help safeguard the viscoelastic properties of tissues in otherwise disused muscles and joints. This approach is particularly important in the sub-acute period after the neural trauma, because in that phase, paresis itself precludes the active work-out of the patient. In addition to this, it can be imagined that a repetitive mobilization of the affected segments could help maintain viable a network of neuronal circuitry that is involved in movement planning and execution, at least by continually providing proprioceptive information and avoiding deafferentation.

In keeping with this latter consideration, our group created a portable mobiliser for the ankle joint, described in [21]. Portability was the fundamental requirement in order to make this device truly available to patients in the acute phase, because they are often bedridden and sometimes cannot even sit upright. Therefore, it was decided that the system should be suitable to be utilized by patients sitting on or lying in bed. This characteristic differentiates the present device from other ones able to produce passive movements of the tibiotarsal joint. The concept was implemented using SMA actuation, because it allows compactness and low weight. For reasons related to the possibility of assessing the central effects of the therapy administered through this device, it was also of interest that the actuator should emit limited electromagnetic noise, in order not to affect electroencephalographic (EEG) measurements. This is also possible using SMA-based technology.

**Figure 1:**
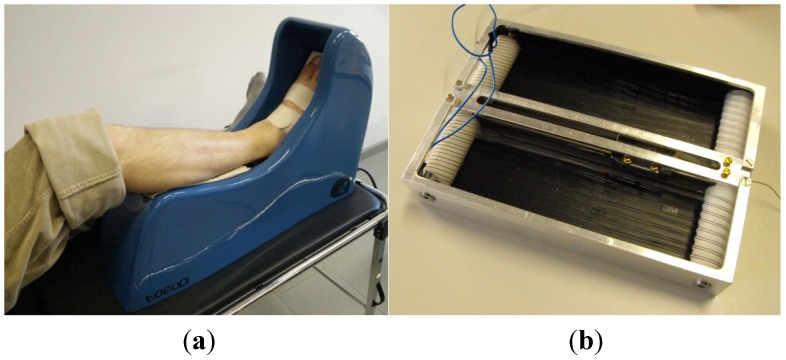
(**a**) The Toe-Up! device for passive ankle mobilization of bedridden patients; (**b**) the shape memory alloy (SMA) actuator used to generate ankle dorsiflexion.

The Toe-Up! device is shown in (Figure 1). It includes a leg rest for the patient’s shank; the foot is strapped to a moving plate that rotates up and down under the combined action of a shape-memory actuator and two pseudoelastic bias springs: the actuator, activated in a cyclic manner by an electronic controller, produces a nominal dorsiflexion of 30°, while the bias springs reset the position in plantarflexion. The actuator is composed of a quadruple arrangement of 0.25-mm NiTi wires. Those wires are electrically connected in series two-by-two, but produce a total force of four (max. 30 N) by pulling in parallel. The particular wire coiling is designed in such a manner that magnetic fields produced by the flowing electric current tend to be cancelled out by antiparallel fields. The actuator is powered from the mains via a transformer/rectifier (56 Vdc) lodged within the device case. Therapy with Toe-Up! was prescribed to seven pediatric patients affected by spastic tetraparesis as a consequence of UML (TBI, traumatic brain injury; AVM, arteriovenous malformation; anoxia). The study protocol comprised two 30-min sessions of passive mobilization a day for 14 days and clinical evaluation pre- and post-treatment. In addition, EEG was recorded from the patients during passive mobilization by Toe-Up! and active movement, to evaluate if any differences in brain cortical (re)activity would arise over the time of treatment. The data collected are currently being analyzed. It can already be anticipated that the device was very well received by the patients and their families and often resulted in an added motivation to work out. The working life of the SMA wire in the actuator depended on the conditions of the patient (particularly on the level of hypertone, which directly affects the intensity of mechanical stress), but it always exceeded at least two weeks. The actuator was always able to produce dorsi-plantarflexion, and no problems were reported about its functioning or safety. Results of preliminary trials at EEG acquisition and analysis, which were carried out on a few healthy subjects, have been published in [22].

**Figure 2:**
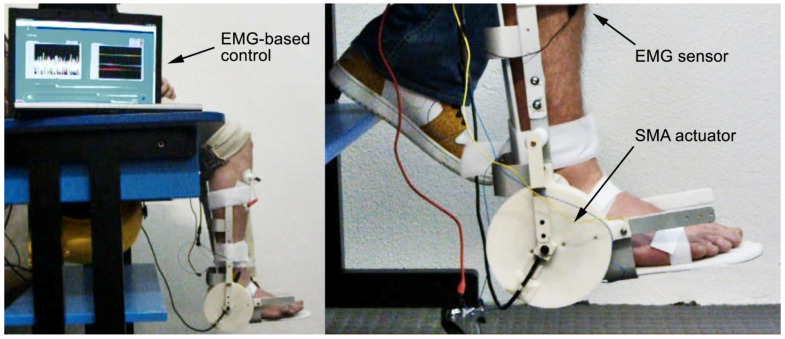
Two views of the EMG-controlled SMA device for assisted ankle exercise.

In order to bridge the gap between the period of time when patients, being paretic, cannot do any active exercise and the later phase in which voluntary control is partially recovered and work-out is recommended, electromyography (EMG) or some other biosignal can be employed to trigger activation of the orthosis. An example of this is described in [23], where a proof-of-concept is provided, with trials on the tibiotarsal joint of healthy volunteers. By setting two adjustable threshold values, it was possible to discriminate between rest, minimal voluntary contraction of the anterior tibialis (TA) muscle and a regular contraction leading to ankle dorsiflexion. Now, if a patient starts regaining some voluntary control of muscular contraction, but this action is not recognized because the contraction is too weak to give rise to a perceivable movement, it could be argued that opportunities for commencing active exercise early might go missed. On the other hand, if minimal contractions are picked up through EMG sensors, these events can be used to trigger passive mobilization by a suitable device. In a way, patients’ involvement would be halfway between active and passive, because movement initiation could be exercised directly by the patient, while the proprioceptive feedback and the reward for that effort would be provided by the device in the form of aided motion. By gradually adjusting threshold values session after session, the therapist could set increasing targets for the patient, ultimately paving the way to active work-out (Figure 2).

### 2.2. Compliant Orthoses for Limb Repositioning

Spastic syndromes, as already mentioned, are characterized by paresis, stiffness, involuntary phasic contractions and jerks of the limbs and, depending on the affected joint, unnatural flexion or extension. Immobility and disuse of the affected joints tend to have adverse consequences, in that holding a static position for a long time can determine a shortening of the muscles and a worsening of contractures and spastic reflexes [24,25]. Orthotic devices can be used to stretch muscles affected by this malformation in an attempt to restore a more physiological neutral posture and increase usable joint range of motion. 

In the practice of standard orthotics, devices are used to hold the affected joint in a fixed position that is closer to the desired one, and muscles are expected to regain in time a more physiological length. The target position can also be changed in a stepwise manner by modifying the orthosis in order to proceed with the treatment. “Dynamic” orthoses are different because they aim at producing muscular remodeling by imposing forces or torques that pull in the desired direction. The target position is not fixed *a priori*, but it is the result of a dynamic balance between the pulling force of the muscles affected by contracture and the force offered by the orthosis. This behavior is much more physiological, because residual movements of the limbs are potentially preserved, and involuntary postural changes are allowed by the device compliance, thus increasing the general comfort. What is truly important is that, thanks to orthosis compliance, immobility and disuse are avoided and so is a major cause of the known negative chronic sequelae of paresis. Under the action of the corrective torque, the muscular lengthening process generally occurs in a slow and gradual manner: in this respect, therefore, the term “dynamic” has to be interpreted just to mean the opposite of “fixed” or “static”. This explanation is given in order to differentiate this usage from that employed in Section 2.3. 

**Figure 3:**
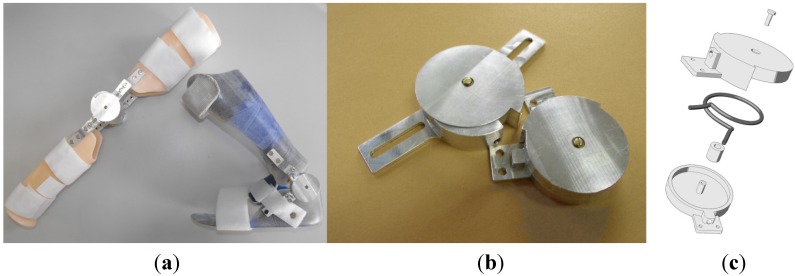
(**a**) Examples of pseudoelastic orthoses; (**b**) pseudoelastic hinge prototypes; (**c**) a drawing of the hinge assembly with the SMA spring (dark grey).

In order to implement these concepts, our group devised a set of hinges [26] that can be used to create compliant orthoses. Inside the hinges are placed two springs made of NiTi, shaped as a capital letter omega (Ω) (Figure 3). This particular shape allows the material to be loaded along its entire length, prevents localized stress concentrations and, ultimately, failures. The spring action is based on pseudoelasticity. The nonlinearity and hysteretic behavior of NiTi-based alloys indeed endow these orthoses with convenient characteristics for this application and solve some inherent problems of dynamic splints with purely elastic elements. In fact, in classic elastic tension or torque elements, the spring-back forces change with elongation; assuming that those elements are preloaded in such a manner as to guide repositioning towards a desired posture, the corrective force applied to the limb will be high at the beginning of the process and will gradually decrease the closer the joint angle gets to the target. Clockwork springs could be used to counter this effect, but they tend to be either weak or bulky. On the contrary, the nonlinear behavior of pseudoelastic SMA, due to the presence of long plateaux at quasi-constant stress, makes it possible to administer a continual therapeutic action even in proximity of the goal and in general for much wider deformation/elongation ranges.

**Figure 4:**
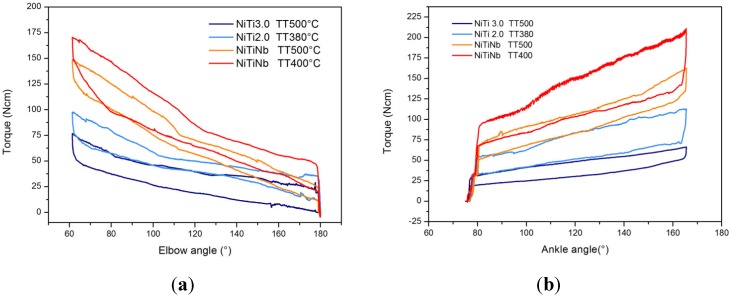
Different elbow (**a**) and ankle (**b**) hinge properties for changing alloy compositions and thermal treatments. Property tuning can be used for customization.

By selecting appropriate thermo-mechanical treatments for the phase of shape setting, it is possible to obtain springs with different plateau stresses and lengths (deformability), and in this manner, alloy properties can be adjusted for different patients’ needs (Figure 4). Hence, the following can be obtained simultaneously: (1) providing a corrective push that is correlated to the biomechanical, biometric and clinical state of the patients, as well as to the likelihood that they will tolerate a given treatment intensity; (2) maximizing acceptability and adherence to prescription times by making the corrective push mild enough and the orthosis sufficiently compliant to involuntary jerks that the pain induced by lengthening on spastic muscles is reduced; (3) avoiding limb fixity, thus improving joint mobility and the chances of a residual use of the limb; (4) avoiding the need to adjust spring preload as posture evolves and the associated burden for caregivers; and (5) self-regulating the strength of the orthotic action in relation to the direction of movement; thanks to SMA hysteresis, the stress during loading is higher than during unloading, so the perceived spring stiffness is higher for actions that are directed against the clinical goal.

The efficacy of this new type of orthoses was compared to that of traditional fixed-angle ones in a cross-over trial carried out on 25 young patients with spastic paresis, mainly in the lower limbs (ankle joint). The results, published in [27], suggest that pseudoelastic orthoses are much more tolerable for patients than traditional ones and are as good at controlling posture. A great advantage of pseudoelastic devices over traditional ones is that, it was found, while traditional devices, due to the immobility and disuse they impose on the affected joint, tend to increase its viscoelastic stiffness during a month’s treatment, pseudoelastic orthoses decrease viscoelastic stiffness over an equal period of time.

The promising results obtained in this study have convinced us to try and expand the functionalities of these dynamic splints and to upgrade the pseudoelastic hinges, in particular for the upper limb.

**Figure 5:**
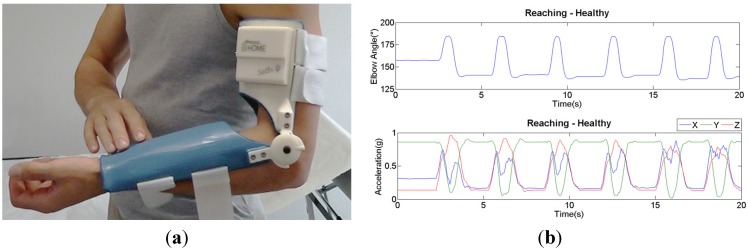
(**a**) A 3D-printed fully-functional prototype of a pseudoelastic sensorized orthosis; (**b**) example of recorded tracings for a healthy subject during a reach-forward task.

New devices were thus developed for producing a therapeutic action, but also for monitoring the evolution of the therapy and recording parameters linked to patient’s activity. The idea is that, taking advantage of the freedom of movement that the pseudoelastic hinges allow, spastic patients with some residual functional control of their affected arm, by virtue of the elbow extension gained through dynamic repositioning and thanks to the improved possibility to reach a larger number of objects and explore the space around them, will start using their limb more often for independent living. The pseudoelastic orthosis would thus be a manner of improving the neutral (average) position of the limb, while its compliance will make sure that flexion-extension will always be an available degree-of-freedom for the execution of motor tasks. On board the orthosis are mounted a potentiometer and a tri-axial accelerometer. Those sensors can continuously log the evolution of elbow joint angle and the accelerations of the proximal arm in time; the tracings are transferred via Bluetooth^®^ technology and made available for real-time reading or off-line processing and viewing by clinicians. Trials are underway on a cohort of 10 post-stroke patients with spasticity in the arm flexors and residual mobility; the purpose of this study is to establish if data collected in the described manner during given standardized tasks can have value for clinically assessing any improvements in the use of the affected arm. Figure 5 shows a completely functional prototype of the sensorized pseudoelastic orthosis; it was designed with a geometry based on individual anatomic images from a healthy volunteer and constructed via 3D printing; the same figure also shows an example of the acquired signals for a standardized motor exercise (reach-forward).

### 2.3. Dynamic Applications and Movement Disorders

Some issues may arise with the use of wearable devices in the rehabilitation of neuromuscular diseases that have truly dynamic features. For instance, patients whose lower extremities are affected (e.g., by drop-foot or equinus) and have a residual capability of independent walking may perceive traditional orthoses as uncomfortable or quite rigid, because, while correcting for the exaggerated plantarflexion during swing, they do not preserve the ability to produce a physiological pattern of walking in all phases of the gait (e.g., plantarflexion is often hindered during propulsion). 

Our group developed and started to test a pseudoelastic AFO (ankle-foot orthosis—similar in concept to the pseudoelastic orthoses described above) in the single case of a young hemiparetic girl with mild spasticity of plantarflexor muscles and equinus foot. Details of that study can be found in [11]. This child was able to walk barefoot and with no aids, but showed incapacity to dorsiflex the ankle joint during the swing phase of the stride cycle. Observing the gait analysis results [11], it is evident that in those conditions, the ankle angle tracing is not physiological and falls out of the normality range. A pseudoelastic orthosis for the ankle joint has been designed and built with a valve on the frontal side of the leg and one on the dorsum of the foot. Straps hold the proximal valve in position, while the patient’s shoe creates a suitable constraint for the other one. In this way, the calf and the sole remain free. The pseudoelastic orthosis was equipped with two *ad hoc* omega-shaped NiTi springs able to support the patient’s foot, leaving the plantarflexion/dorsiflexion motion free. At the moment of the test, the patient was asked to walk first barefoot, then with a traditional AFO (which was her original prescription) and, finally, with the pseudoelastic device. Gait was recorded in all three different conditions by an optoelectronic system. The results suggest that the pseudoelastic splint allows a much more physiological walking pattern with respect to the traditional one or the barefoot condition; the preparation of heel-strike is improved; the plantarflexion phase that is needed for optimal propulsion before toe-off is preserved; and so is the spontaneous EMG activity during swing. By comparison, plantarflexion (and thus, an efficacious propulsion) is completely prevented, and swing-phase EMG is silent with the traditional AFO. A final consideration on the material is that the two springs, not being made of simple steel, but of pseudoelastic NiTi, provide a quasi-constant response: plantarflexion is allowed, and even though bringing the ankle into plantarflexion produces an extra deformation in the spring, this deformation does not correspond to an increase in the force required to carry out the movement (as would be the case with linear materials). Furthermore, as already stated, the material properties are tunable, which means that the action of this type of orthosis can be adjusted to the specific needs of each patient, also following the prescriptions of the clinicians. 

This first dynamic application in the context of a spastic syndrome gave us an inspiration to experiment with treating of other neurologic disorders characterized by dynamic components. In particular, there are some diseases classified under the definition of movement disorders, which affect both the young and adults and are very disabling. For these patients, very important aspects of life, such as social relationships and independence, are often compromised; the available solutions in some cases have relevant negative drawbacks, e.g., pharmacological treatments may have limited efficacy in adults and a number of systemic adverse effects in children; those issues may lead to changing very frequently the type of drugs or the dosage and long delays for establishing the correct treatment. On the other hand, surgery is invasive and, thus, appropriate for very severe clinical pictures in otherwise untreatable adults [28] and often not recommended for children. Orthoses and devices for damping or at least controlling the involuntary movements caused by most movement disorders, like tremors, dystonias and dyskinesias, have been developed in the past [3], but they are often bulky, heavy and totally uncomfortable solutions, usable just for research purposes or some spot-wise applications, but hardly for a continuous use in daily life circumstances: e.g., robotic systems are often effective, but they are too uncomfortable to wear or even carry around in ordinary life situations; sometimes, even when therapeutic devices have actually been thought out considering the portability and usability by the subject (e.g., collars), they often neglect the social aspects of their visual impact and sometimes fail to meet important biomechanical aspects, as avoiding load shielding of muscles and bones. For some of the mentioned disorders, psychological components have a particular relevance, and they should be taken into account in the design process in order to ensure that the patient will have a benefit from using the device and will actually wear it and use it; in other cases, solutions are too much of a constraint on the affected limb, limit the possibilities of voluntary motion and may ultimately lead to a disuse of the muscles and structures involved, possibly worsening in some manner the specific and the general conditions of the patient. 

In light of these considerations, it is evident that different and innovative approaches are needed, in order to develop wearable devices to control motor disorders. Our research is focusing on movement disorders that hardly find other valid solutions: in particular, axial tremor and cervical dystonia in adults and hyperkinetic disorders in children. For these specific applications with high dynamic characteristics, we are designing some devices to control and dampen unwanted involuntary movements, by exploiting the peculiar properties of NiTi alloys in terms of pseudoelasticity, internal friction and mechanical hysteresis. These new devices will have to be compliant and light and as comfortable and “transparent” as possible. In order to meet this goal, some basic criteria ought to be kept in mind: (1) proper knowledge of the pathology is essential, including its patient-specific features in terms of range of motion, frequency, amplitude, direction of involuntary movements and individual strategies to control the symptoms; (2) a plan is needed for how to tune specific material properties in the most efficient and precise way to customize the devices to individual patients’ needs; and (3) the material-limb interfaces used to transmit the SMA action to the affected segments must be carefully designed in order to be comfortable even in dynamic conditions and as aesthetic and discreet as possible. We are currently working on those three aspects and can share the following considerations: psychological and sensory mechanisms do appear to be fundamental in reducing the symptoms of MD; the use of high-energy-density SMA as core elements in the development of therapeutic devices could help concentrate into very small, light and acceptable parts the dynamic repositioning and movement-conditioning functionality of the device; the expected action is likely to have the mild-force characteristic that was effective in connection with UML orthoses and should be as well tolerated. We have experience in the patient-specific adjustment of SMA behavior; in this case, rather than concentrating only on stresses, strains and optimal pseudoelasticity, we shall also consider the damping properties, which could play a role; at the moment, we are investigating the possibility of tuning these characteristic in relation to the dynamic features of patients’ voluntary and involuntary movements in order to interfere minimally with the former, while controlling the latter. Finally, due to the dynamic nature of MD, the design of orthosis interfaces with the human body will have to consider limb shape and size, both at rest and during movement, in order to increase the efficiency of SMA action transfer (useful to reduce SMA size) and maximize comfort.

### 2.4. Amagnetic Devices and Movement Guides 

In the study of diseases that affect limbs and have a neurologic origin, diagnostic instrumentation, such as magnetic resonance imaging (MRI), magnetoencephalography (MEG) and other bioimaging techniques, are primary tools that provide information, such as the precise determination of damage, the investigation of structural and functional reorganization in the central nervous system and the peripheral neuromuscular one and also the assessment of the natural evolution or changes related to the application of some specific therapy. All of these technologies impose strict constraints in terms of material compatibility and acceptable noise generated by external electromagnetic sources that may affect the quality of the acquisitions. For the purpose of evaluating neuromuscular rehabilitation and investigating the brain correlates of motor actions in neurological disorders, it would be invaluable to possess a set of tools able to provide repeatable sensorimotor stimulation or to standardize the parameters of active and passive movements during bioimaging acquisitions: considering that standardization and repeatability may play an essential role in increasing signal-to-noise ratios in repeated-measures experiments, thus enabling more efficient and less time-consuming protocols in bioimaging, the intent of building devices totally compatible with MRI, MEG, *etc.*, appears to be of fundamental importance for these studies. It is evident that only some classes of materials can be employed for designing limb guides or devices for limb mobilization, due to the severe dimensional and electromagnetic constraints imposed by those technologies. A partial solution to avoid this problem is to place actuators and all incompatible parts outside the shielded rooms, but this requires long transmission lines that could pick up environmental noise and corrupt the quality of the data collected. 

SMAs can be considered interesting materials for the development of this kind of device because of their high energy density (actuators and spring-back elements can be very compact) and magnetic compatibility with diagnostic instrumentation (MRI and MEG). Exploiting the intrinsically non-magnetic behavior of the NiTi alloy and designing a specific arrangement for the SMA wire, our group has developed different types of actuators that are virtually amagnetic. A special wire arrangement is required to counter the negative effect of using electric current to activate NiTi, *i.e.*, the fact that, due to the current, an undesired electromagnetic field arises around the wire. By coiling the wire in such a way that for every loop, there is a counter-loop, closely-spaced, coaxial and antiparallel to the first, magnetic emissions can be strongly abated by mutual cancellation of the fields generated by the accelerated electric charges flowing in opposite directions. This principle was implemented in different ways obtaining linear actuators as explained in [10] and rotary ones as shown in [29]. Both designs enabled successful neuroscience studies on brain reactivity to passive ankle mobilization in healthy subjects, respectively [20,30] and [20,31]. 

More recently, we are utilizing a pseudoelastic elbow guide for functional MRI (fMRI) and EEG studies in post-stroke patients with spasticity. The guide was created with an aluminum frame, and it is equipped with mechanical stops for directing the movement on a reproducible path and limiting movement stroke to a reproducible range; all parts not made of aluminum were created by a 3D printer in ABS*plus*. Thanks to the quasi-constant push generated by the spring-back of pseudoelastic NiTi elements, this device also provides help to the patient in executing elbow extension, which is most problematic in this neurological condition. The compactness of NiTi springs in this application (low number of turns for a given angular stroke, compared to other materials, e.g., spring steel) allowed us to make the device profile very reduced, so the whole system can fit both size-wise and emission-wise into the gantry of a standard MRI machine with the patient wearing standard head coils. The design was published in [32].

## 3. Material and Methods 

### 3.1. Materials

For the present applications, a number of different binary and ternary NiTi-based alloy compositions (Ni-rich NiTi, Ti-rich NiTi, NiTiNb) have been used. In particular, several Ni-rich compositions and NiTiNb were employed for the pseudoelastic devices: starting from batches of wire between 3 and 6 mm in diameter, suitable samples (generally around 2–3 mm in diameter) were prepared by cold drawing. Those work-hardened samples were shape-set at appropriate temperatures and for appropriate durations to reach good and stable pseudoelasticity. The works in [27] and [33] fully discuss the aspects connected to the choice of alloy composition and thermomechanical treatment for our pseudoelastic applications. For the thermally-activated SME applications, we generally used commercial trained Ti-rich NiTi wire for actuation applications (*A_f_* approximately 90 °C, mostly 0.25 mm in diameter), in order to make the devices easier to replicate by others. 

### 3.2. Background on SMA Phenomenology

Shape memory alloys (SMAs) are a heterogeneous class of metal alloys; the main groupings are ferromagnetic and non-ferromagnetic alloys, but only the non-ferromagnetic ones have practical applications. Among the non-ferromagnetic SMAs, two classes of compositions have been employed practically: Cu-based and NiTi-based, but the NiTi-based ones are the most widespread due to their superior characteristics in the majority of applications. Ni-Ti is a quasi-stoichiometric intermetallic compound with the predominant feature of undergoing an athermic reversible martensitic transformation between a cubic (B2) parent phase (called austenite) and a monoclinic (B19’) one (called martensite). This phase transformation in the metallic lattice is at the base of interesting macroscopic effects, such as the shape memory effect (SME) and pseudoelasticity (PE). The direct B2–B19’ transformation can be obtained by either cooling or introducing mechanical strains. Cooling the alloy in the B2 structure to a temperature lower than a characteristic point (*M_s_*) induces exothermal formation of B19’ in twinned ordering, without any macroscopic change in shape; on the other hand, subjecting B2 to strains in excess of approximately 1%–1.5% provokes the reversible movement of atomic layers and the formation of an un-twinned (or de-twinned) version of B19’. Conversely, starting with a martensitic structure, the heating the alloy above a characteristic temperature (*A_s_*) sets an endothermal transformation from B19’ to B2; straining a twinned B19’ structure produces a de-twinned B19’. For thermally-driven processes, transformations can be considered complete below *M_f_* (direct transition B2–B19’) and above *A_f_* (reverse transition B19’–B2). In the mechanically-driven formation of detwinned martensite, stresses for initiating the process are proportional to the alloy temperature (Clausius–Clapeyron’s law). The precise values of characteristic temperatures *M_s_*, *M_f_*, *A_s_* and *A_f_* are functions of Ni content and also depend, more subtly, on thermomechanical processing. In particular, increases of one part in the thousands in Ni atomic concentration can produce drops by tens of degrees Kelvin in characteristic temperatures. Coming to the practical exploitation of these effects, because the detwinning stress of initially twinned martensite is low compared to the detwinning stress for the B2 phase (the latter is stable at higher temperature), NiTi is more deformable in martensitic state. By detwinning B19’, it is possible to obtain recoverable strains of up to 10%; recovery is achieved through heating above *A_f_* and the consequent reverse transformation to B2. This is called the SME. Thanks to SME, a weight heavy enough to induce B19’ detwinning at a temperature lower than *M_f_* can be lifted during heating-induced strain recovery at a temperature appropriately higher than *A_f_*. SME is indeed a means of making solid-state actuators. PE is a different aspect of the same phenomenon. For alloys having an *A_f_* lower than room temperature, the initial state before straining is B2. With a sufficient level of stress, detwinned martensite can be formed by deformation. Strains up to 10% are readily recoverable upon removing the loading stress, because, the working environment being at a temperature higher than *A_f_*, B2 is re-formed immediately. In this case, both the direct (loading) and inverse (unloading) transformations occur at constant stresses. The two plateaux are separated by mechanical hysteresis, and the area of the hysteresis loop corresponds to energy lost by structural viscosity in the process of loading and unloading. Fatigue life in SMA depends strongly on the levels of stress and maximal strains. In particular, strains of 10% are incompatible with cyclic applications, for which 4% can be considered an upper limit, unless a high number of cycles is required, in which case, strains as low as maximum 1% or less would be appropriate, depending on stress intensity. Overheating can lead to increased accumulation of plastic deformation and early failure, this obviously being more relevant an issue for typical SME than PE applications, e.g., [34,35]. 

### 3.3. Tunability and Optimization of Characteristics

The properties of SMA are tunable, *i.e.*, by changing alloy composition or applying suitable thermo-mechanical treatments, material characteristics, such as the transformation temperatures, the height and length of the stress plateaux, and to some extent, the hysteresis, the cycling stability, the internal friction, *etc.*, can be adjusted to the final application. This opportunity offered by SMA can be exploited to modify material behavior and meet specific clinical requests, as well as the needs of the one patient for whom a certain therapeutic device is made. Some of our studies (e.g., [27]) took full advantage of that possibility, by customizing every single device to patients’ characteristics, such as age, severity, affected joint, tolerance, pain, *etc.* Let us consider a wearable device application, like an orthosis: in practical terms, it can be imagined that a certain number of optimized processes can be utilized to produce SMA elements with as many different final properties, each of which may be suitable for a sub-group of patients with a set combination of the mentioned characteristics (age range, severity range, *etc*.). In this manner, *ad hoc* devices could be prescribed for each sub-group, thus improving tolerability and outcomes. Process identification can be obtained in various manners [34,36]. For pseudoelastic applications, we devised a simple method [33] based on the monitoring of SMA shape setting in real time through the use of an electric probe. In particular, it was found that, for a given treatment temperature, if shape setting is stopped at a precise time point corresponding to the start of a slow decrease in specimen resistance, shape setting, pseudoelasticity and cycling stability are simultaneously optimized.

## 4. Final Remarks and Conclusions 

The nonlinear and easily-adjustable characteristics of SMA make this class of materials a very interesting resource in the development of new devices and new therapies for neurologic conditions. This paper discussed a number of applications, in which SMA provides added functionality, allows customization and improves tolerability and outcomes in the clinical management of UML patients. The work done so far on MD is preliminary, but based on previous experience and initial observations, we believe that the dynamic properties of SMA could help also in the design of alternative therapies for otherwise untreatable movement disorders. Future work will go into expanding evidence about the clinical validity of the proposed devices and clarifying the extents of their applicability and indication. 
